# Pulsed Laser Deposition of SWCNTs on Carbon Fibres: Effect of Deposition Temperature

**DOI:** 10.3390/polym13071138

**Published:** 2021-04-02

**Authors:** Călin Moise, Lidar Rachmani, Geanina Mihai, Oana Lazar, Marius Enăchescu, Naum Naveh

**Affiliations:** 1 Center for Surface Science and Nanotechnology, Politehnica University of Bucharest, 313 Splaiul Independentei, 060042 Bucharest, Romania; calin.moise@cssnt-upb.ro (C.M.); geanina.mihai@cssnt-upb.ro (G.M.); oana.lazar@cssnt-upb.ro (O.L.); marius.enachescu@cssnt-upb.ro (M.E.); 2 Department of Polymer Materials Engineering, Shenkar College of Engineering and Design, Ramat Gan 5252626, Israel; lidarrahmani@gmail.com; 3 Academy of Romanian Scientists, 54 Spaiul Independentei, 050094 Bucharest, Romania

**Keywords:** surface treatments, interface, carbon fibre, pulsed laser deposition, SWCNTs

## Abstract

Single wall carbon nanotubes (SWCNTs) were grown on either sized or desized carbon fabric in a self-designed reactor by Pulsed Laser Deposition (PLD). The uniqueness of the PLD system lies, among other things, in the ability to keep the substrate at a low temperature, compared to the 1100 °C needed for the SWCNTs synthesis, thus, rendering it undamaged. Samples were placed at different positions on a cold finger (CF), where a temperature gradient develops, in the range 25–565 °C. The chemical composition and morphology of desized and surface treatments, as well as SWCNTs grown on carbon fibres, were verified by Scanning Electron Microscopy (SEM) equipped with Energy Dispersive X-Ray Spectroscopy (EDX), while the quality of SWCNTs was proven by confocal micro-Raman Spectroscopy and High-Resolution Scanning Transmission Electron Microscopy (HR-STEM). Fibres covered with SWCNTs by PLD were characterized using contact angle and the surface free energy was calculated. A micro-droplet pull-out test was used to evaluate the effect of SWCNTs over interfacial properties of a carbon-epoxy composite. A 20% increase in interfacial shear strength (IFSS) was observed by deposition at 290 °C, compared to the commercial carbon fibre sizing. The carbon fibres kept their tensile properties due to the low deposition temperatures.

## 1. Introduction

Composite materials comprising fibres and a polymeric matrix owe their properties to those of the components and their interaction at the interface. Some properties are very much biased by the characteristics of the interface, which can be regarded as a third phase, thus, the term interphase. Toughness, strength perpendicular to the fibres, and fatigue are among these properties. 

Efforts to strengthen and toughen the composite have been reported; these include chemical or physical surface treatments [1,2,3,4,5] as well as dispersion of Carbon Nanotubes (CNTs) [6], graphene [7], and other nanoparticles. Chemical functionalization such as wet oxidation produces active polar groups on the fibre surface [5]. Other forms of oxidation include dry gaseous oxidation such as air plasma treatments, which also produce active polar chemical groups on the fibre surface [8,9]. Active groups formed on the fibre surface are then covalently or physically bonded to the matrix during curing of the system. Moreover, these polar chemical groups increase the surface polarity of the fibre, resulting in improved wetting of the fibre by the matrix. Physical modification of the fibre includes chemical etching such as low power plasma, which, in turn, increases the specific area of the fibre [9]. Various forms of carbon nano-particle deposition have been studied. These include (CNTs) [10], Graphene Oxide (GO) [11], and modifications of these particles. These physical alterations have an important role in increasing the specific area of the fibre and acting as a bridging structure protruding into the matrix and increasing the interfacial strength between the fibre and the matrix. 

The treatments affect the Surface Free Energy (SFE) of the fibre. Chemical functionalization will likely increase the polar SFE of the fibre, whereas physical altering of the fibre may increase the dispersive SFE of the fibre. A higher polar SFE is generally preferable due to the fact that many matrices are polar in nature, and, thus, better wetting of the fibre will occur, which is crucial for increasing the Interfacial Shear Strength (IFSS).

CNTs have been grown on the surface of carbon fibres by chemical vapor deposition (CVD). Yao et al. [12] applied CVD on carbon fibres at 700 °C for 1 h. Catalyst concentration was shown to control growth, while fibre desizing and oxidation were shown to affect CNT distribution on the fibre surface. Thermal CVD was also used by Zhang et al. [13] to grow MWCNT on carbon fibres. Temperatures in the range 700–800 °C affected CNT growth, however, deposition at such high temperatures impaired fibre tensile strength.

Synthesis of Single Wall Carbon Nanotubes (SWCNTs) has been reported by Enachescu’s group since 2014 [14]. In a review book chapter [15], the advantages of laser ablation over other methods were described in detail for the synthesis of carbon nanomaterials and the particularities of the self-designed reactor. Composite polymers with SWCNTs were prepared to increase the performances of solar cells [16,17]. The reinforcement induced by SWCNTs in composites or polymers is still a hot subject for researchers [18].

In this work, nano-structured interphases are developed for carbon/epoxy composites by depositing SWCNTs on either sized or desized carbon fibres, to attain both strength and toughness. The effect of the deposition temperature is investigated, then, SWCNTs-coated fibres are furthermore characterized, among others, from a morphological and micromechanics standpoint. Relatively low deposition temperatures are enabled with the self-designed reactor.

## 2. Materials and Methods

### 2.1. Materials

FT300B 3k 40B carbon fibres (Toray Industries Inc., Tokyo, Japan) were taken from a unidirectional (UD) fabric of 3k tows produced by CIT Composite Materials S.R.L. (Legnano (MI), Italy). The probe liquids were ethylene glycol (EG), a polar liquid with known polar and dispersive surface tensions, 19 mJ/m^2^ and 29 mJ/m^2^, respectively, and n-hexadecane, a nonpolar liquid with a dispersive surface tension of 27 mJ/m^2^. The target used for SWCNTs synthesis containing 0.6% Ni, 0.6% Co, and 98.8% C (atomic percentages) was produced following a recipe developed by Enachescu’s group [19]. 

### 2.2. Sample Preparation

**Figure 1:**
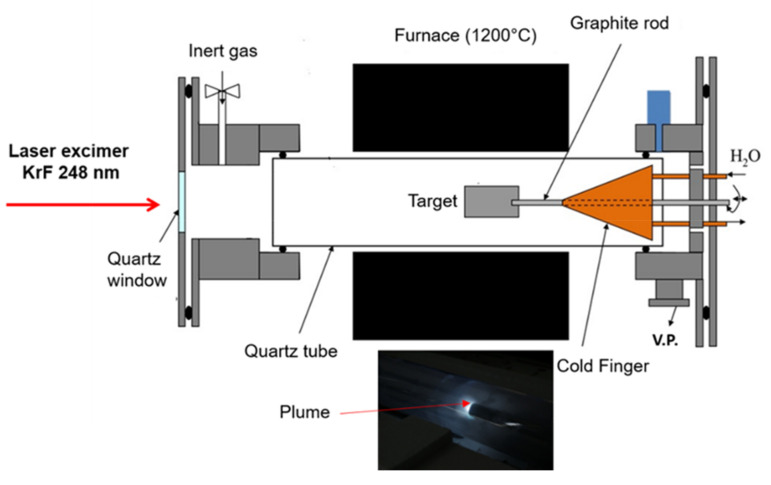
Self-designed reactor for Pulsed Laser Deposition (PLD)—patent pending, in inset the plume ejected from target.

Commercial carbon fibres were used as is or after desizing. The desizing process is described elsewhere [11]. Desizing was confirmed by SEM, FTIR, and XPS. Sized and desized fibres were placed in a self-designed Pulsed Laser Deposition (PLD) reactor, schematically described in Figure 1.

The KrF excimer laser used for the ablation was COMPex Pro 205, produced by Coherent Inc. (Santa Clara (CA), USA). It has a pulse duration of 20 ns and a wavelength of 248 nm. In our study, we used a laser energy of 600 mJ and a 30 Hz repetition rate.

All the ablation parameters like laser energy, laser repetition, temperature, and pressure, were selected based on previous optimization studies [15]. The oven temperature, pressure, and total ablation time were: 1100 °C, 500 torr, and 60 min, respectively. The laser beam passes through a UV transparent quartz window and enters the quartz tube hitting the target, thus, initiating the target ablation. The target was rotated during ablation with constant speed to get a uniform ablation. The plume is ejected to the left (see inset in Figure 1). The inert gas, which enters from the left side of the reaction chamber flows laminarly through the quartz tube to the 1100 °C heated area where the reaction takes place, thus, transporting the ablation product toward a semi-conical shape, 300 mm in length, copper Cold Finger (CF), where it is deposited as a black soot. The CF was cooled down using 20 °C water supplied by a chiller. The inert atmosphere and the transportation of the ablated material to the CF were maintained by using Argon gas at a 70 L/h flow rate. 

In all the experiments, SWCNTs synthesized by PLD were deposited directly onto the carbon fibres. 

During oven operation at 1100 °C a thermal gradient develops along the CF surface since its tip is much closer to the oven centre as can be seen in Figure 1. In a preliminary study, fibres (sized and desized) were located directly on the CF’s surface in different positions; consequently, the deposition temperature exhibited a moderate variation. 

For high temperature depositions, 100–565 °C, the desized fibres were suspended in different positions above the CF, thus, avoiding the thermal contact with it. The desized fibres received the temperature from the oven, according to their position. The temperatures were monitored in each position using a thermocouple. 

### 2.3. Characterization 

#### 2.3.1. Micro-Raman and STEM Characterization

Characterization of SWCNTs was done by Confocal micro-Raman (HORIBA Ltd., Kyoto, Japan) using an excitation wavelength of 532 nm (green laser) and by High Resolution Scanning Transmission Electron Microscopy (HR-STEM) Hitachi HD-2700 (Hitachi High-Tech Corp., Tokyo, Japan). 

#### 2.3.2. Surface Microstructure by SEM

Scanning Electron Microscopy (SEM) was performed with a Hitachi SU8230 unit (Hitachi High-Tech Corp., Tokyo, Japan) equipped with EDX Oxford detector-analyser (Oxford Instruments PLC, Oxford, UK) in order to study the fibres covered with SWCNTs. 

#### 2.3.3. Contact Angle and Surface Free Energy

Single fibres were carefully picked from the tow and bonded to a paper frame. A droplet of probe liquid was dispensed with a micropipette. To obtain an accurate measure of the length and peak height of the droplets, an Olympus optical microscope was used. A magnification of 200× was found to be ideal for these measurements. 

#### 2.3.4. Micro-Droplet Pull-Out Test

**Figure 2:**
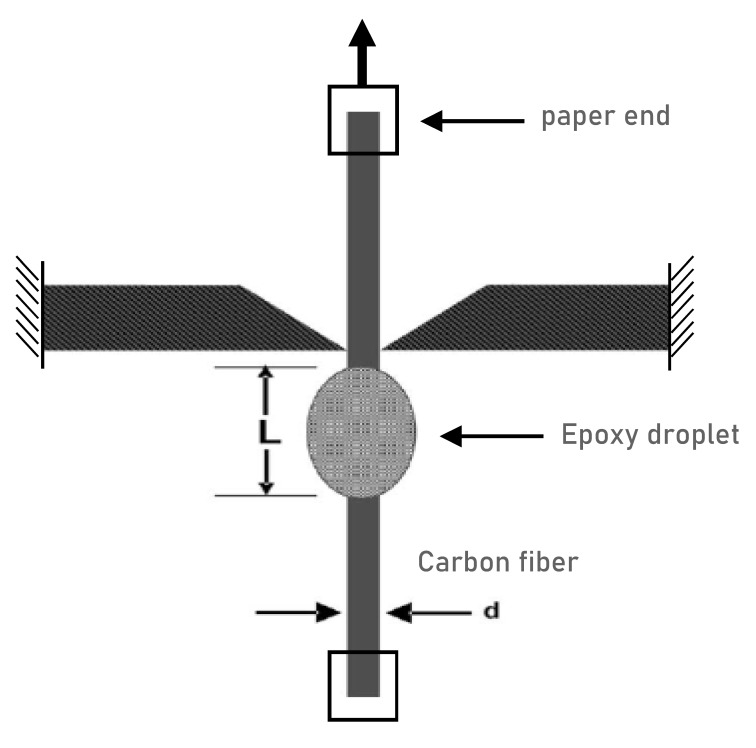
Micro-droplet pull-out test setup.

The fibre was bonded to a paper frame as before. An epoxy droplet, 70 microns diameter in average, was applied on the fibre and cured up to 160 °C. Then, the frame was halved, and one paper end was attached to a moving sample holder in a force machine. A pair of knives was placed on each side of the fibre, close to the droplet, to constrain its position. The paper end was pulled at 0.1 mm/min until the droplet de-bonded by shear. The setup of this test is shown in Figure 2.

#### 2.3.5. Tensile Test

Single carbon fibres were tested in tensile mode with an Instron machine at 0.1 mm/min. Specimens were prepared as in Section 2.3.4. Tensile strengths were reported.

## 3. Results

### 3.1. SWCNTs Characterization

**Figure 3:**
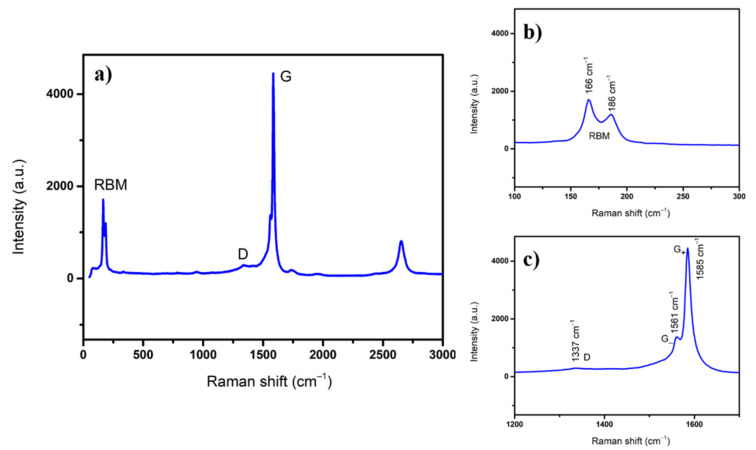
(**a**) Typical Single Wall Carbon Nanotubes (SWCNTs) Raman spectra; (**b**) radial breathing mode (RBM) band and (**c**) D and G bands.

Confocal micro-Raman spectroscopy was used for SWCNTs characterization. Raw material was collected from the edge of CF where the deposition temperature was 25 °C. In Figure 3a, a typical Raman spectrum of SWCNTs acquired at room temperature is shown. It is well known that SWCNTs present a special fingerprint in Raman spectra, namely, the radial breathing mode (RBM) band on lower frequency, a disorder band called D band, and a graphitic G band at higher frequencies [20]. 

From the RBM band, the value of SWCNTs diameters can be determined via an empirical Equation (1) [21]. As can be seen in Figure 3b, there are two peaks in RBM corresponding to SWCNTs diameters: 1.28 and 1.46 nm. G band splits into two bands called G_-_ and G_+_ as shown in Figure 3c; the ratio I_D_/I_G_ = 0.07 indicates high quality of synthesized SWCNTs.

d = c_1_/(ω − c_2_) (1)

where:

ω—frequency of vibrations in the radial direction [cm^−1^]
c_1_, c_2_—constants [cm^−1^];
c_1_ = 215 [cm^−1^]
c_2_ = 18 [cm^−1^]
d—the diameter of the nanotubes [nm]

**Figure 4:**
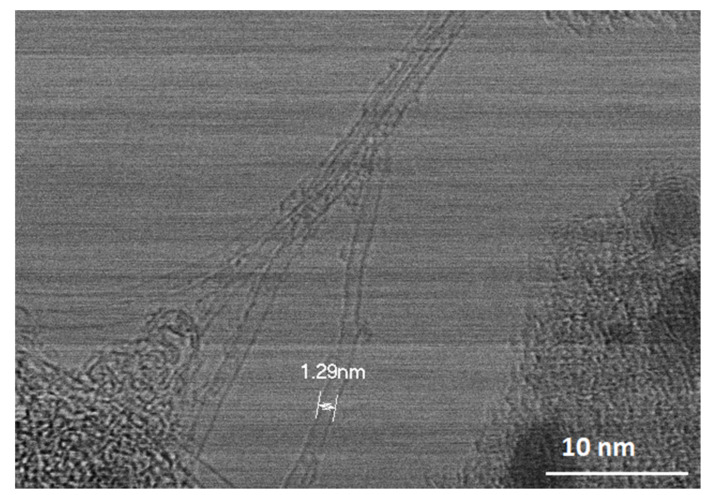
Measurement of individual SWCNT diameter by HR-STEM.

HR-STEM images reveal SWCNTs with diameters around 1.3 nm, as shown in Figure 4. This is in good agreement with the values calculated via micro-Raman and in a previous study regarding the diameter distribution of SWCNTs, where an average diameter of 1.35 nm was found [22]. In conclusion, high quality SWCNTs are produced via PLD. 

These high quality SWCNTs were deposited directly on the carbon fibres at different temperatures as described in sample preparation. 

### 3.2. Fibres Covered by SWCNTs 

**Figure 5:**
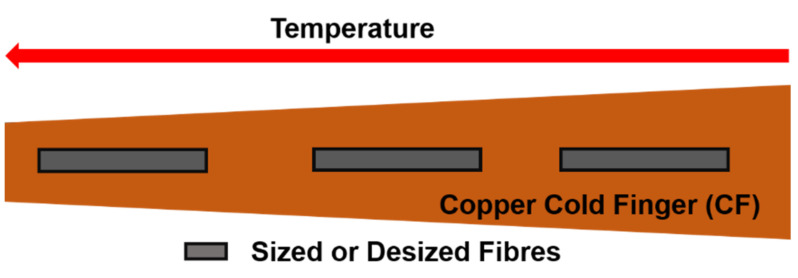
Schematics of the sample’s positions on the Cold Finger for moderate temperature variation.

In a preliminary study, desized fibres were placed directly onto the CF at different positions. A temperature gradient develops, such that the temperature is higher at its tip and lower at its base, as was described in Sample Preparation. The fibres’ positions on the Cold Finger are indicated in Figure 5 shown below.

**Figure 6:**
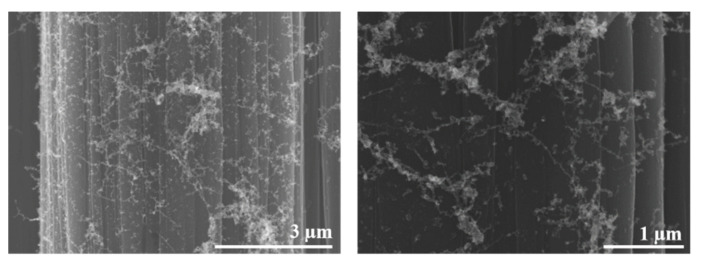
SWCNTs network on desized carbon fibres at low nodular growth rate.

Temperature affects how SWCNTs are distributed along the fibres, as well as the SWCNTs growth rate. At low to mid temperatures, depending on the position of the fibre in the tow, a fine, rather even, covering of the fibre surface was obtained. In Figure 6, SWCNTs seem to arrange in a two-dimensional network connected by nodules. For a nucleation and growth mechanism, deposition probably started at the nodules, and extended in between due to the low growth rate at these rather low temperatures.

**Figure 7:**
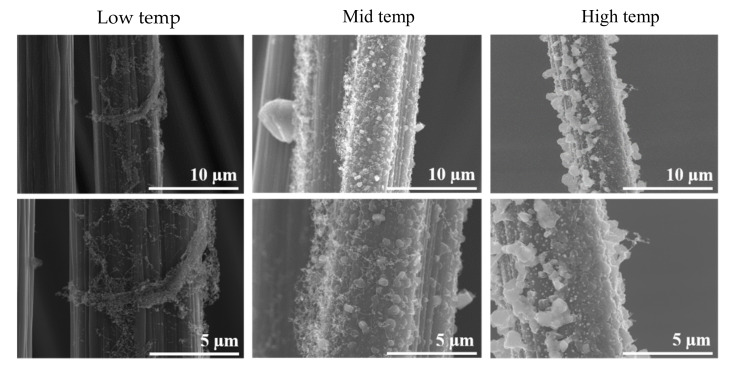
SWCNTs nodule and cluster formation on desized carbon fibres.

As the deposition temperature increases, the nodules become larger and turn into clusters. The micrographs in Figure 7 reveal the effect of the deposition temperature. Low temperature resulted in uneven deposition with few desized fibres being coated, yet finer CNT nodules, 100 nm and less. Higher temperatures led to the formation of larger CNT nodules or clusters, in the order of 0.5 µm.

**Figure 8:**
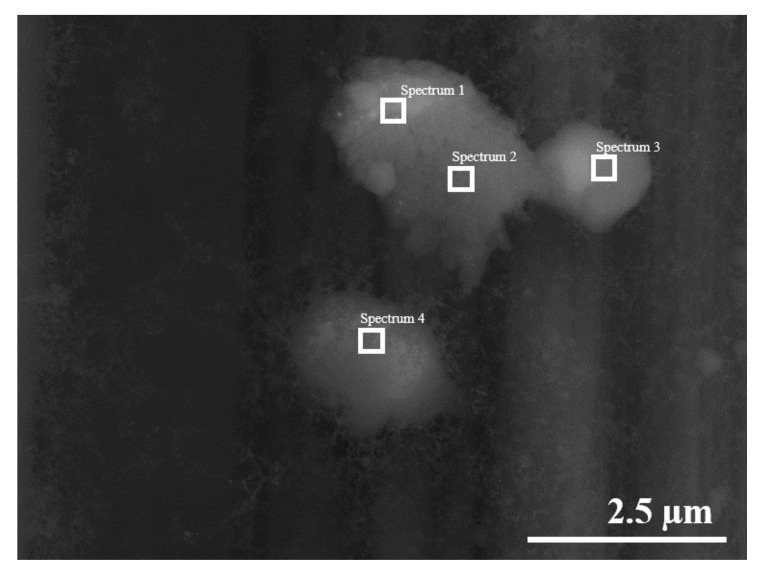
Determination of carbon clusters composition by EDX.

The carbon clusters were confirmed by EDX, typical results are given in Figure 8 and Table 1.

Next, a comparison was made between sized and desized fibres after exposure to mid-range temperatures. 

**Figure 9:**
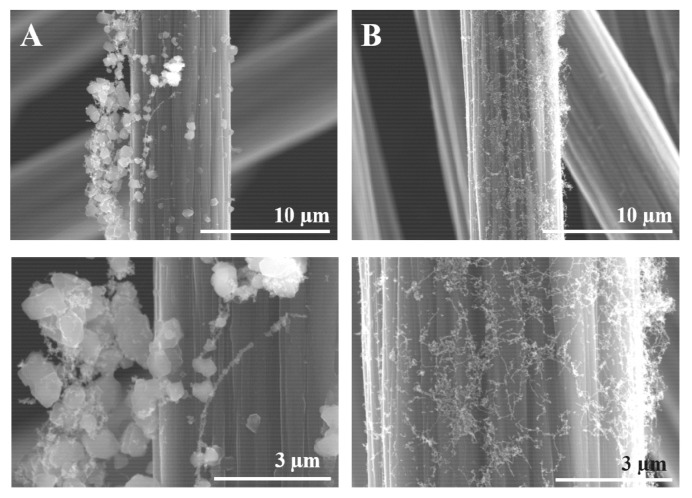
Effect of sizing on PLD of SWCNTs on carbon fibres: (**A**) desized fibres, and (**B**) sized fibres.

Figure 9A shows that the desized fibres have a coarse, uneven pattern of SWCNTs clustering, whereas Figure 9B shows that sized fibres were evenly coated with finer clusters.

For a nucleation and growth mechanism, sizing seems to favour nucleation, while the desized fibres rather promote carbon cluster growth. 

**Figure 10:**
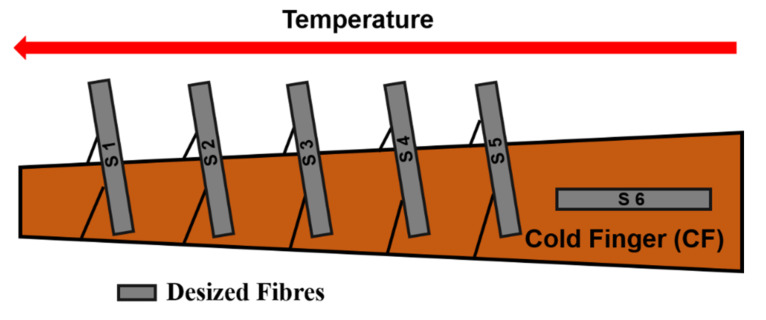
Schematics of the sample’s positions on the Cold Finger for deposition at various temperatures.

Finally, desized fibres were exposed to several high temperatures by controlling the position while monitoring the temperature, as can be seen in Figure 10. 

This way, deposition took place at a wide range of temperatures, from 25 °C to 565 °C. Desized fibres are expected to withstand the relatively high exposure temperatures, while the sizing of commercial fibres would degrade and loose mass.

Samples synthesized for this study are indexed in Table 2.

**Figure 11:**
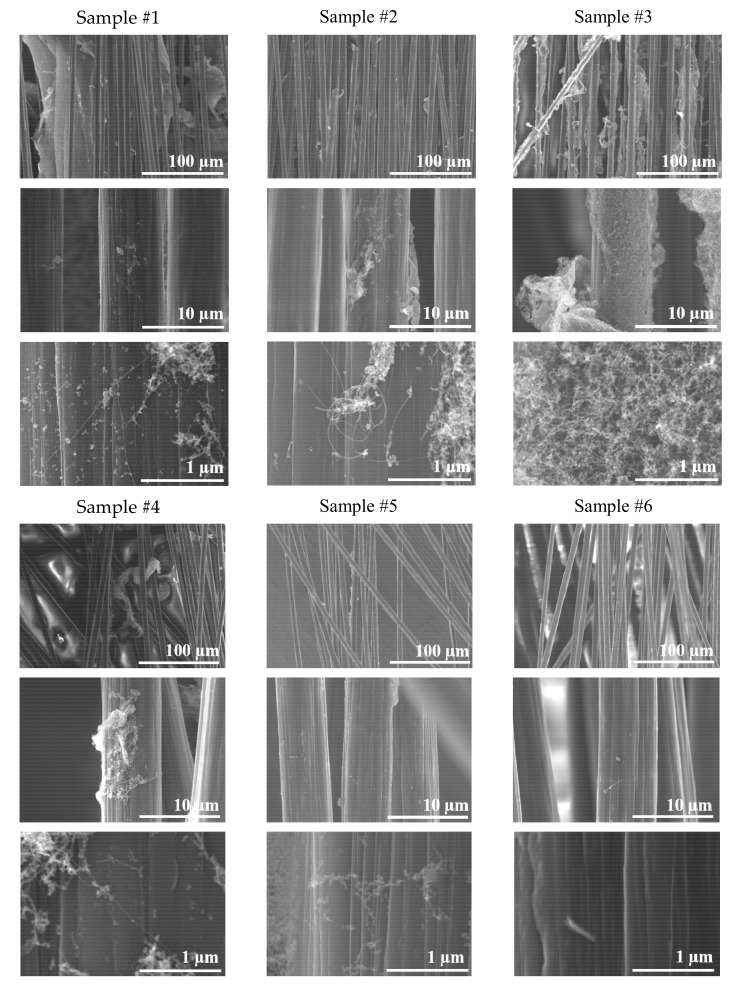
SEM micrographs of desized carbon fibres at various temperatures, PLD exposures, several magnifications.

SEM micrographs in Figure 11 show that the best deposition took place at 290 °C, on Sample 3, with a rather uniform coverage of the fibres. As can be seen, all the other samples, obtained at higher or lower temperatures, present some amount of SWCNTs on the fibres, but the coverage is not uniform. Deposition at 25 °C is much finer. Although uneven, a subtle coating of SWCNTs appears on extensive areas on the carbon fibres. 

**Figure 12:**
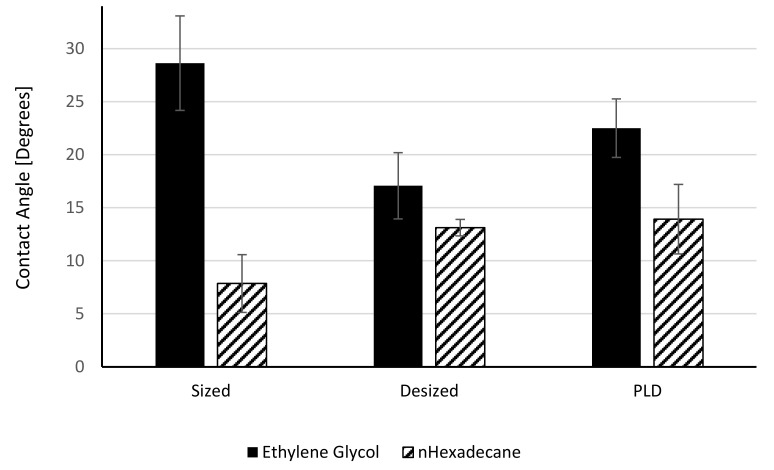
Contact angles of desized fibres after SWCNTs deposition.

**Figure 13:**
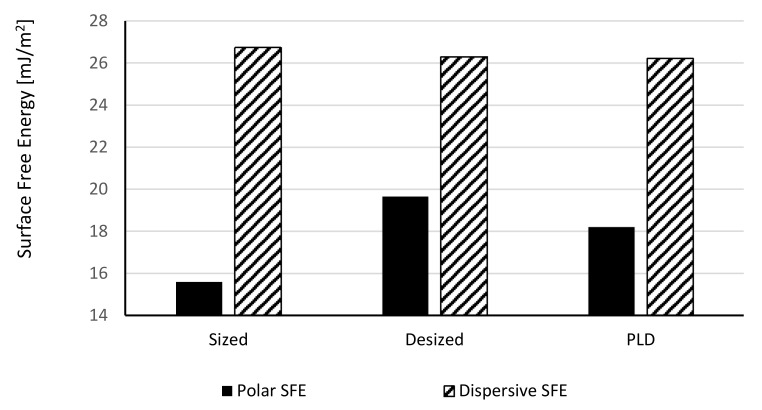
Surface Free Energy (SFE) of desized fibres after SWCNTs deposition.

Contact angles and surface free energies of the fibres are shown in Figure 12. The SFE calculation is based on the Owens–Wendt equation [23], which in turn is based on Young’s equation [24]. It assumes smooth surfaces. We can elaborate on the validity of this equation for desized and SWCNTs coated fibres. The macroscopic, measurable contact angle depends not only on the thermodynamics of a smooth surface, but also on the effect of roughness. Desized carbon fibres were shown to exhibit larger surface areas than commercial fibres, by 10%. Desizing also appears to leave functional groups on the fibre surface [25]. Nevertheless, a SWCNTs network deposited on the carbon fibre surface increases the roughness at the micrometre to nanometre scale and may induce some porosity. Models like Wenzel’s equation [26] and Cassie–Baxter’s equation [27] have been proposed to take into consideration the effects of roughness and heterogeneities. Wenzel’s model is usually applied for low contact angles (θ_W_ < 90°) and predicts a reduction of contact angle for “wetting” liquids due to surface roughness, whereas Cassie–Baxter’s model is considered for large contact angles (θ_CB_ > 90°) and predicts an increase of contact angle for non-wetting liquids. With a polar liquid, desized and PLD samples show contact angles lower than the sized (commercial) fibres, and PLD samples show values somewhat higher than those of the desized fibres. This translates into a stronger polar component of the SFE of desized fibres in Figure 13, while the dispersive component remains unaffected. The results for the desized fibres can be explained by the increased surface area and the increased polarity. Thus, desized fibres seem to follow the Wenzel model. However, an increased polarity of the PLD fibres cannot be explained by the composition of the coating, characterized in Table 1 by low oxygen content. The fine structure of the SWCNTs coating seems to increase the surface roughness and overall surface area of the fibre and may explain the reduction in contact angle of a polar liquid, compared to the commercial fibre. The opposite behaviour is seen with a nonpolar liquid. The increase in n-hexadecane contact angle indicates the oleophobic nature of the SWCNTs. SWCNTs are mostly carbon and, thus, hydrophobic and oleophobic, and their interaction with a nonpolar liquid leads to higher contact angles, as shown for n-hexadecane. Thus, in this case SWCNTs-coated carbon fibres seem to follow the Cassie–Baxter model, which predicts an increase in contact angle. We conclude that the wetting behaviour of SWCNTs-coated carbon fibres is complex, and either wetting or de-wetting may happen, depending on the morphology of the coating and the polarity of the wetting agent.

**Figure 14:**
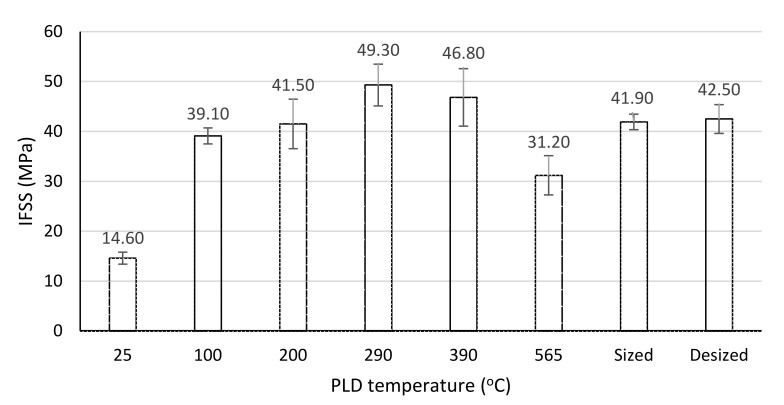
Interfacial shear strength (IFSS) of desized fibres PLD-treated at different temperatures. Values for sized (commercial) and desized fibres are shown for comparison.

Microdroplet pull-out tests of SWCNTs-coated fibres exhibit a bell-shaped dependence on temperature with a maximum at 290 °C. See Figure 14. Such behaviour can be explained by the nucleation and growth model. SWCNTs nucleation is more effective at the lower temperatures, but the SWCNT clusters are very small and the coating is uneven, leaving extensive surface areas uncovered. Intermediate temperatures allow for significant nucleation and growth, and a rather even coating is achieved. Higher exposure temperatures hamper growth, possibly due to cleavage of carbon–carbon linkages, and by 565 °C there is no deposition. SEM micrographs in Figure 11 seem to support this explanation.

Near 20% increase in Interfacial Shear Strength (IFSS) is obtained for deposition at 290 °C, compared to the sized (commercial) fibre. Although optimization was not done, these results show the potential to enhance the properties of the interface and increase the IFSS of carbon/epoxy composites. Interestingly, desizing did not affect the IFSS. Previous work indicates that desizing is not thorough, and certain functional groups, including urethane moieties, remain and contribute to matrix bonding [25].

**Figure 15:**
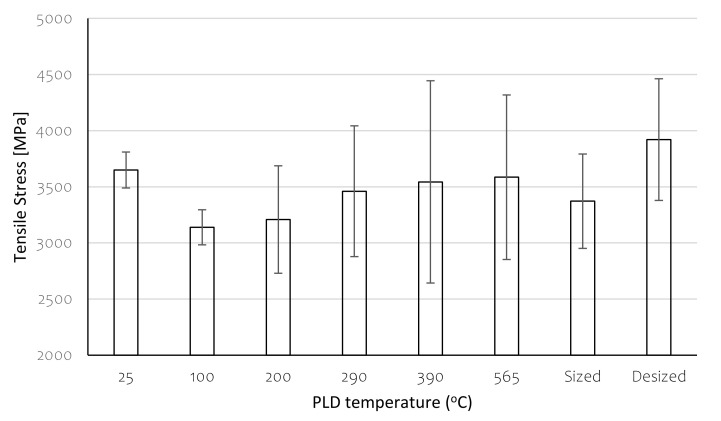
Tensile strength of PLD-treated fibres. Values for sized (commercial) and desized fibres are shown for comparison.

Carbon fibres after exposure to the various temperatures and SWCNT deposition are shown to keep their original Tensile Strengths in Figure 15. This is due to the relatively low exposure temperatures enabled by this innovative PLD process. These conclusions are valid despite the relatively large standard deviations for the PLD-treated fibres, which clearly evolved during desizing. 

## 4. Conclusions

SWCNTs were deposited on carbon fibres by PLD in a self-designed reactor. SWCNTs quality was proved by micro-Raman spectroscopy and HR-STEM.

SEM images prove the successful deposition of SWCNTs onto carbon fibres at several temperatures. EDX was involved for chemical composition.

Deposition of SWCNTs on the carbon fibres at certain conditions seems to reinforce the carbon/epoxy interface. In this study, a 20% increase in interfacial shear strength (IFSS) was observed by deposition at 290 °C, compared to the commercial carbon fibre sizing, without any effect on the tensile properties of the carbon fibres. SWCNTs deposition also affects the surface free energy and contact angles of liquids and may promote wetting or de-wetting depending on the polarity of the liquid.

## Figures and Tables

**Table 1:** Composition of the formed SWCNTs clusters.

Scheme 1.	Spectrum 1		Spectrum 2	Spectrum 3		Spectrum 4
C	96.23		95.70	96.05		97.67
O	3.77		4.30	3.95		2.33
Total	100		100	100		100
**Statistics**		**C**			**O**	
Max		97.67			4.30	
Min		95.70			2.33	
Average		96.41			3.59	
Standard Deviation		0.87			0.87	

**Table 2:** Temperatures at which the analysed samples were obtained.

Sample Name	Temperature (°C)
Sample #1	565
Sample #2	390
Sample #3	290
Sample #4	200
Sample #5	100
Sample #6	25

## Data Availability

The data presented in this study are available on request from the corresponding author.
